# A Comparative Study of Pyrolysis Liquids by Slow Pyrolysis of Industrial Hemp Leaves, Hurds and Roots

**DOI:** 10.3390/molecules26113167

**Published:** 2021-05-25

**Authors:** Ayobami Salami, Jorma Heikkinen, Laura Tomppo, Marko Hyttinen, Timo Kekäläinen, Janne Jänis, Jouko Vepsäläinen, Reijo Lappalainen

**Affiliations:** 1 Department of Applied Physics, University of Eastern Finland, P.O. Box 1627, 70211 Kuopio, Finland; jorma.a.heikkinen@gmail.com (J.H.); laura.tomppo@uef.fi (L.T.); reijo.lappalainen@uef.fi (R.L.); 2 Department of Environmental Science, University of Eastern Finland, P.O. Box 1627, 70211 Kuopio, Finland; marko.hyttinen@uef.fi; 3 Department of Chemistry, University of Eastern Finland, P.O. Box 111, 80101 Joensuu, Finland; timo.kekalainen@uef.fi (T.K.); janne.janis@uef.fi (J.J.); 4 School of Pharmacy, University of Eastern Finland, P.O. Box 1627, 70211 Kuopio, Finland; jouko.vepsalainen@uef.fi; 5 SIB Labs, University of Eastern Finland, P.O. Box 1627, 70211 Kuopio, Finland

**Keywords:** pyrolysis liquid, slow pyrolysis, industrial hemp, chemical characterization, NMR, GC-MS, APPI FT-ICR MS, volatile compounds, nonvolatile compounds, economic assessment

## Abstract

This study assessed the pyrolysis liquids obtained by slow pyrolysis of industrial hemp leaves, hurds, and roots. The liquids recovered between a pyrolysis temperature of 275–350 °C, at two condensation temperatures 130 °C and 70 °C, were analyzed. Aqueous and bio-oil pyrolysis liquids were produced and analyzed by proton nuclear magnetic resonance (NMR), gas chromatography–mass spectrometry (GC-MS), and atmospheric pressure photoionization Fourier transform ion cyclotron resonance mass spectrometry (APPI FT-ICR MS). NMR revealed quantitative concentrations of the most abundant compounds in the aqueous fractions and compound groups in the oily fractions. In the aqueous fractions, the concentration range of acetic acid was 50–241 gL^−1^, methanol 2–30 gL^−1^, propanoic acid 5–20 gL^−1^, and 1-hydroxybutan-2-one 2 gL^−1^. GC-MS was used to compare the compositions of the volatile compounds and APPI FT-ICR MS was utilized to determine the most abundant higher molecular weight compounds. The different obtained pyrolysis liquids (aqueous and oily) had various volatile and nonvolatile compounds such as acetic acid, 2,6-dimethoxyphenol, 2-methoxyphenol, and cannabidiol. This study provides a detailed understanding of the chemical composition of pyrolysis liquids from different parts of the industrial hemp plant and assesses their possible economic potential.

## 1. Introduction

The industrial hemp plant (*Cannabis sativa.* L.) is one of the cannabis varieties that is grown as an agricultural crop. Industrial hemp is an annual, non-psychoactive plant selected for its low concentration of the psychoactive compound, delta-9-tetrahydrocannabinol (Δ-9-THC), i.e., not more than 0.3% on a dry wet basis. Industrial hemp is characterized as a second-generation lignocellulosic biomass [1] and is mainly composed of cellulose (39–50%), hemicelluloses (18–25%), and lignin (21–24%) [2,3,4]. 

Hemp is a fast-growing crop that provides high quantity biomass in a short time with 10–15 t·ha^−1^ of hemp dry matter yields, a short vegetation period of 3–4 months, and a rapid growth up to 4 m in height. The short rotation nature of this crop and its fast growth are closely linked to the potential of using this biomass [5]. According to the European Industrial Hemp Association’s (EIHA) report published in 2017, hemp cultivation and its market have increased considerably and undergone a remarkable development since the 1990s due to the unique properties of this plant, making it suitable for many applications. Over 51 hemp species have been developed since 2013, compared to 12 species cultivated prior to 1995 [6].

Industrial hemp has two main commercial products, namely fibers and seeds. The main by-product is hurd from the production of fibers and seeds, but also the leaves or roots of the plant can be collected. Most of the leaves of hemp plant grown for fiber are returned to the field to serve as soil mulch. As a wide range of hemp varieties are good candidates for phytoremediation [7], extractives in the woody biomass such as hemp plant stalks and roots can be extracted and exploited for chemical, medicinal, and other uses. Roots are major contributors to the total biomass in woody and wood plants and they play critical roles in carbon sequestration [8,9]. Medical uses of hemp roots have been studied, e.g., *Cannabis sativa.* L. roots have been used to treat fever [10], difficult childbirth [11], gastrointestinal activity [12], sexually transmitted disease [13], inflammation [14], tumors, arthritis, and joint pain, as has been previously reviewed [15]. Despite a long history of medicinal use, the roots of cannabis plants have been mostly ignored in modern medical research and thermochemical processes.

The pyrolysis of biomass is an irreversible thermochemical conversion method that involves the thermal decomposition of biomass in the absence of oxygen to obtain three product states, i.e., solid char, condensable gases, and non-condensable gases [16,17]. The classification of pyrolysis methods such as slow pyrolysis, fast pyrolysis, and gasification depends on, e.g., temperature, heating rate, reaction, and residence time. A key factor enhancing the environmental sustainability of slow pyrolysis of biomass is the production of biochar and its various applications as well as the production of pyrolysis liquids that are rich in organic compounds [18,19].

Lignocellulosic biomass distillates produced by fractional condensation in the slow pyrolysis process can be categorized into two categories: a water-based solution (pyroligneous acid) containing acetic acid, methanol, acetone, methyl acetate, etc., and a bio-oil or tar containing phenols, levoglucosan, triterpenes, and others. The processes underpinning the slow pyrolysis of biomass have been extensively studied, especially process parameters, process stages, heating rate, operation, and condensation temperatures [17,19].

Several pyrolysis studies of industrial hemp seeds and hurds have been carried out, especially on a small laboratory scale [20,21]. The need to investigate the chemical composition of lignocellulosic biomass pyrolysis liquids has led to the extensive use of various modern analytical techniques including Fourier-transform infrared spectroscopy (FTIR), nuclear magnetic resonance spectroscopy (NMR), gas chromatography-mass spectrometry (GC-MS), liquid chromatography (LC), liquid chromatography-mass spectrometry (LC-MS), two-dimensional gas chromatography (2D GC-MS), and thermogravimetric analysis (TGA). GC-MS has played an important role in revealing the chemical composition of volatile compounds of lignocellulosic biomass bio-oils. 2D GC-MS has been successfully applied to separate some higher MW compounds present in bio-oil since this technique has a superior separation capability than conventional GC-MS due to its two columns with different polarities, which can be exploited at the same time [22,23,24]. Fourier transform ion cyclotron resonance (FT-ICR) mass spectrometry (MS) is an important technique for analyzing complex mixtures due to its ultra-high mass resolution, especially targeting the least volatile heavy ends of pyrolysis liquids [25,26]. Of the chemical characterization techniques mentioned above, NMR has played a crucial role in elucidating the chemical composition and concentration of compounds in pyrolysis liquids from lignocellulosic biomass [23,24]. Several biomasses including different varieties of industrial hemp bio-oils have been investigated and analyzed with the above approaches [27,28,29].

Despite all the studies mentioned above, there is limited information regarding the concentration of different compounds in distillates from the whole industrial hemp plant, i.e., the leaves, hurds, and roots. Furthermore, it would be desirable to perform a chemical characterization of pyrolysis liquids, especially with respect to hemp roots. The understanding of the chemical composition of pyrolysis liquid is important for better application, separation, and purification. In this study, we conducted a comprehensive comparative investigation of the chemical composition of volatile and non-volatile compounds of the aqueous (water-based solution) and bio-oil fractions obtained after the slow pyrolysis of hemp leaves, hurds, and roots, especially using quantitative NMR. The second objective was to determine the chemical compositions of the volatile and non-volatile compounds by applying GC-MS and (APPI) FT-ICR MS, respectively. To our knowledge, this is the first scientific study to have investigated and compared the chemical composition of the aqueous and bio-oil fractions of industrial hemp leaves, hurds, and roots especially with several liters of slow pyrolysis reactor scale. We describe new data for the concentration of compounds in different hemp pyrolysis liquid samples and compare the chemical composition of distillates from different sections of the industrial hemp plant, leaves, hurds, and roots. Our working hypothesis was that the concentrations of biomolecules in distillates from leaves, hurds, and roots would be significantly different.

## 2. Materials and Methods

### 2.1. Sample Preparation

Futura 75 is a widely used industrial fiber hemp which is grown in Finland. Industrial hemp seeds of the variety of Futura 75 were planted in Kivennapa, Juankoski, Finland (63.0697° N, 27.9989° E) at the end of spring 2016 and were manually harvested in autumn 2016. Hemp harvest yield in our field experiment on dry basis was: leaves 6.4 t·ha^−1^, stem (fibre and hurds) 34.3 t·ha^−1^, and roots 7.8 t·ha^−1^. The pretreatment of the samples was carried out manually, separating the leaves from the stalks. Hemp hurds were obtained by manually peeling the fiber from the hemp stalk and the roots were collected and cleaned to remove soil. All samples (leaves, hurds, and roots) were stored at 5 °C prior to further processing. Each sample was manually compressed into steel tubes with a hydraulic press using a pressure of 10 MPa. The liquids released during the compression were weighed to evaluate the mass balance. The moisture content (dry weight) for each of the sample was LV 106%, HR 135%, and RT 130%.

### 2.2. Slow Pyrolysis Process

**Figure 1:**
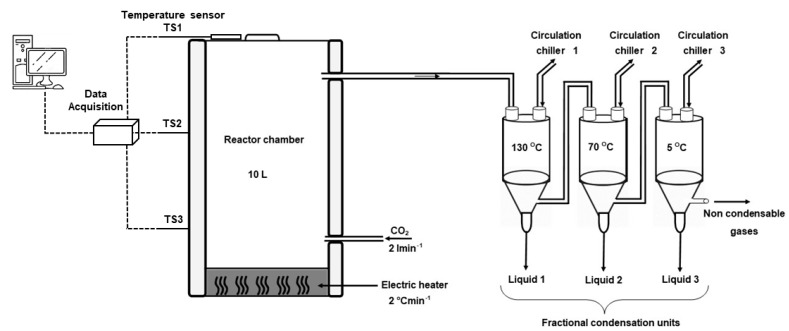
Schematic diagram of stepwise slow pyrolysis process.

The slow pyrolysis runs (at low temperature) were carried out in a batch pyrolysis reactor of a 10 L stainless steel reactor with a diameter of 30 cm. An electric heater was used to heat the reactor in a controlled manner with a typical heating rate of 2 °C min^−1^ and a carrier gas of CO_2_ flow rate of 2 L min^−1^. In this study, slow pyrolysis liquids were categorized into three stages based on the operating temperatures which were selected for drying stage DS (22–135 °C), torrefaction stage TS (135–275 °C), and pyrolysis stage PS (275–350 °C). There were three liquid collection points in the pyrolysis process set at condensation temperatures of 130 °C, 70 °C, and 5 °C. The average retention times for the drying, torrefaction, and pyrolysis stages were 20 h, 20 h, and 5 h, respectively. Hemp pyrolysis liquids selected for analysis were liquid fractions obtained at an operating temperature range of 275–350 °C and condensation temperatures 130 °C and 70 °C for each raw material. The criteria for the sample selection were based on identifying phenols (product of lignin degradation), especially the most abundant phenols that can be obtained at low pyrolysis temperatures. Lignin is a recognized rich source of valuable green chemicals and distillates by the thermochemical process [30,31,32]. The schematic diagram of the stepwise slow pyrolysis process is shown in Figure 1.

### 2.3. Nuclear Magnetic Resonance (NMR) and Chemical Characterization

NMR spectroscopy method was used in the identification of the structure of chemical compounds by detecting proton (^1^H) and carbon-13 (^13^C) nuclei and their environment within the molecules. ^1^H NMR spectroscopy was used to obtain the general profile of the compounds. The selected samples were measured by using a Bruker Avance III HD 600 MHz NMR-system (Billerica, MA, USA) operating with liquid nitrogen cooled multinuclear Z gradient 5 mm cryoprobe at the ^1^H frequency of 600 MHz equipped with an automated sample changer. The chemical shift table of ^13^C NMR was used to determine the carbon environment in a molecule.

NMR samples were divided into two categories based on the condensation temperatures 130 °C and 70 °C. The first category, C1 (bio-oil), consisted of leaves (LV1), hurd (HR1), and roots (RT1) pyrolyzed at temperature 275–350 °C and condensed at a temperature of 130 °C. The second category, C2 (aqueous), consisted of LV2, HR2, and RT2 pyrolyzed at temperature 275–350 °C and condensed at 70 °C. The sample mass of LV1, HR1, and RT1 used for the NMR measurements was 18.8 mg, 27.2 mg, and 20.8 mg, respectively, and was dissolved in 475 μL of deuterated methanol MeOD (CD_3_OD). The sample volume of LV2, HR2, and RT2 was 50 μL; it was dissolved in 400 μL of deuterated methanol MeOD (CD_3_OD). Note: The difference in the sample mass of LV1, HR1, and RT1 was due to its oily form, which is difficult to measure to accurate using a pipette in contrast to LV2, HR2, and RT2 in aqueous form. All samples were prepared in 5 mm NMR tubes. The volume of TSP 25 μL of 1.5 mM of trimethylsilyl propionic-2,2,3,3-d4 acid sodium salt (TSP) was added to all the samples as an internal reference standard at chemical shift 0 ppm.

#### 2.3.1. 2D NMR Spectroscopy

The structural elucidation of the most abundant compounds in the pyrolysis liquid was carried using 2D NMR techniques such as correlation spectroscopy (COSY), heteronuclear single quantum correlation (HSQC), and heteronuclear multiple bond correlation (HMBC). 2D NMR homonuclear correlation spectroscopy COSY (^1^H-^1^H) was used to determine correlations between neighboring protons in the identified compounds. HSQC was used to determine ^1^H-^13^C single bond correlations, where ^1^H directly bonded to ^13^C. HMBC was used to identify correlations between ^1^H-^13^C that are two or more bonds apart from each other [33]. The coupling constants (*J*) indicate the number of neighboring protons and their distance (typically two or three bonds away) in the carbon backbone [34].

#### 2.3.2. Concentration Calculation

The measured ^1^H NMR spectra were phased manually. The baseline correction was carried out using the Cubic Spline Baseline Correction routine in the Bruker Topspin software. Measurements were analyzed by using Bruker BioSpin GmbH TopSpin 4.0.9 software (Billerica, MA, USA). The procedure for the calculation of the concentration of compounds in the pyrolysis liquids is as follows: (1) Spectrum phase adjustment; (2) Baseline correction; (3) Checking of chemical shifts with TSP referenced at 0 ppm; (4) TSP peak integration and calibration to 9.000 (TSP has 9 ^1^Hs); (5) Integration of other peaks; (6) Concentration calculation of the identified compound. The parameters used in the concentration calculation of the identified compounds were a volume of TSP 25 μL and 50 μL of the sample. From the NMR spectrum, the peak area of the identified compound was integrated (integrated peak area) and the number of protons corresponding to the integrated peak was considered as shown in Equation (1).

(1) $$Conc\left(mM\right)=\left(\frac{Integrated\;peak\;area\;/\;Number\;of\;protons}{Sample\;volume\;/\;TSP\;volume}\right)\times TSP\;Conc.\;\left(mM\right)$$

It is important to notice that the concentration of one of the hemp hurd distillate fractions used in this study was determined in our previous study [24]. This distillate fraction is included in this study for proper comparison of each part of the hemp plant using the same measurement parameters and to determine how accurate NMR analysis could be in determining the concentration of compounds of distillates.

### 2.4. GC-MS

Samples were diluted with methanol at a 1:10 ratio and they were analyzed in a gas chromatograph (Agilent 7890B, Agilent Technologies Inc., Santa Clara, CA, USA) and a mass spectrometer (Agilent 5977A). An Agilent HP-5 silica capillary column (30 m × 0.25 mm, film thickness 0.25 µm) was used. Split injection in a 1:20 ratio was used and the injector temperature was 250 °C. The temperature program was 40 °C, hold for 8 min, increase 5 °C/min to 180 °C and then increase 10 °C/min to the final temperature of 250 °C, maintained for 20 min. Helium was used as the carrier gas. The SCAN method was used with an atomic mass unit from 33 to 400. The identification of compounds was accomplished by retention times and GC-MS data library (NIST02, National Institute of Standards and Technology, Gaithersburg, MD, USA). The relative abundance of the total ion chromatogram areas of the compounds was calculated. The method was used for quick screening of the distillates and to support the other analytical methods used in the study.

### 2.5. FT-ICR Mass Spectrometry

All experiments were performed on a 12-T Bruker Solarix XR FT-ICR instrument (Bruker Daltonics, Bremen, Germany), equipped with an Apollo-II atmospheric pressure ion source. Positive-ion APPI was used as an ionization technique in all of the measurements due to its higher selectivity toward neutral, aliphatic, and aromatic compounds. The samples were dissolved in a toluene-methanol mixture (30:70, *v*/*v*) to a concentration of ~100 µg/mL. The samples were introduced into the ion source by a syringe pump (at a flow rate of 10 mL/h) through a heated nebulizer operating at 400 °C under a nitrogen sheath gas. For each spectrum, 100 co-added 8 MWord time-domain transients were summed, full-sine apodized, and zero-filled once to provide the final 16 MWord magnitude-mode data in an *m*/*z* range of 100 to 2000. The instrument control and data acquisition were performed by Bruker ftmsControl 2.1 software. The initial spectral post-processing was done with Bruker DataAnalysis 5.0 SR1 software, including internal mass re-calibration with a calibration list, made in-house for conventional bio-oil samples. The data were then transferred to PetroOrg IS 18.0.3 software (Omics LLC, Tallahassee, FL, USA) for molecular formula assignments and data visualization. Only the peaks with a signal-to-noise ratio (S/N) ≥ 6 were taken into account. In the assignments of molecular formulae, monoisotopic compositions were limited to ^12^C_1–100_, ^1^H_1–200_, ^14^N_0–6_, ^16^O_0–30_, ^32^S_0–2_, with a double bond equivalent (DBE) of 0–50 and a mass error of ≤1.0 ppm.

## 3. Results and Discussion

The thermal degradation by slow pyrolysis of industrial hemp leaves, hurds, and roots produced water-based solutions and bio-oil fractions. NMR, GC-MS, and (+) APPI FT-ICR MS have been proven to be useful and reliable analytical tools in the chemical characterization, identification, and quantification of chemical composition. As hypothesized, the concentrations of biomolecules varied significantly (by more than an order of magnitude) between distillates from leaves, hurd, and roots. For example, hurd contained the highest concentration of alcohols, esters/sugars, and phenols, however leaves were rich with aromatics (cannabinoids). The chemistry of slow pyrolysis at low temperatures and the results of each analytical technique are discussed separately below.

### 3.1. Slow Pyrolysis Product Yields

Liquid condensates (bio-oil and water-based solutions), biochar, and non-condensable gases were obtained from the slow pyrolysis process of industrial hemp leaves (LV), hurds (HR), and roots (RT) carried out at a low temperature range of 22–350 °C. A total number of 27 raw pyrolysis liquids were obtained from three slow pyrolysis runs (9 liquid fractions obtained per process) as presented in Table 1, which also describes the process run details and the mass balance of the samples. The main purpose of Table 1 is to show the mass balance of each sample with respect to the product yields of the process.

The results in Table 1 indicate that the yield distribution of the liquids at the drying stage was affected mainly by the moisture content of the samples. However, the liquid yields at the torrefaction and pyrolysis stages were affected by the type of chemical constituents and density of the samples. The mass loss percentage, which consists mostly of the non-condensable gases, was LV 22%, HR 27%, and RT 21%. The biochar mass percentage was 29% for LV, HR 30%, and 29% for RT, i.e., about the same. The pyrolysis liquid mass percentage was LV 47%, HR 43%, and RT 48%. Although the pyrolysis process parameters were optimized for biochar production, the liquid yields were higher and biochar yields correspondingly lower. The higher amount of pyrolysis liquids released after drying stage for wet materials suggests that moisture was still left in the sample after drying stage. The findings of this study are in accordance with the observations of [24], revealing that wet biomass such as green hemp provides higher pyrolysis liquids yield when compared to biochar while dry biomass produces higher biochar yield when compared to the liquid. The results revealed that the type of biomass used has an effect on the biochar and liquid yields and by optimization of the process it would be possible to steer the process to maximize biochar or liquid yields. In order to optimize the yields and take into account environmental and economical impacts, the type of biomass, heating rate, residence time and operating temperature are important parameters to consider.

### 3.2. Chemistry of Slow Pyrolysis

The chemistry of slow pyrolysis begins with drying which is carried out below 130 °C. The degradation of chemical components of lignocellulosic biomass begins around 130–150 °C due to the evaporation of chemically bound water. The decomposition of hemicelluloses starts to take place above 180 °C [35]. The reaction is endothermic when the temperature is below 200 °C. The process becomes exothermic around 220–270 °C and the major compounds produced are acetic acid, formic acid, and carbon dioxide [36]. Decomposition of cellulose starts around 240 °C and it happens at different stages, starting with dehydration, then decarboxylation, carbonization at 300 °C, and bio-oil is formed around 320 °C [37]. Cellulose experiences secondary reactions by cracking the vapors into secondary biochar, tar, and gases. Lignin decomposition occurs over a broad range of temperatures from around 200 °C up to 500 °C. Extractives decomposition also occurs over a broad range of temperature from around 150–400 °C [36,38].

### 3.3. Chemical Characterization

We compared the industrial hemp pyrolysis liquids and identified differences in the chemical composition and the concentration of the major chemical compound groups. Out of the 27 raw liquids obtained, 6 liquids were selected, measured, and analyzed in detail using ^1^H NMR, GC-MS, and APPI FT-ICR MS.

#### 3.3.1. ^1^H NMR

**Figure 2:**
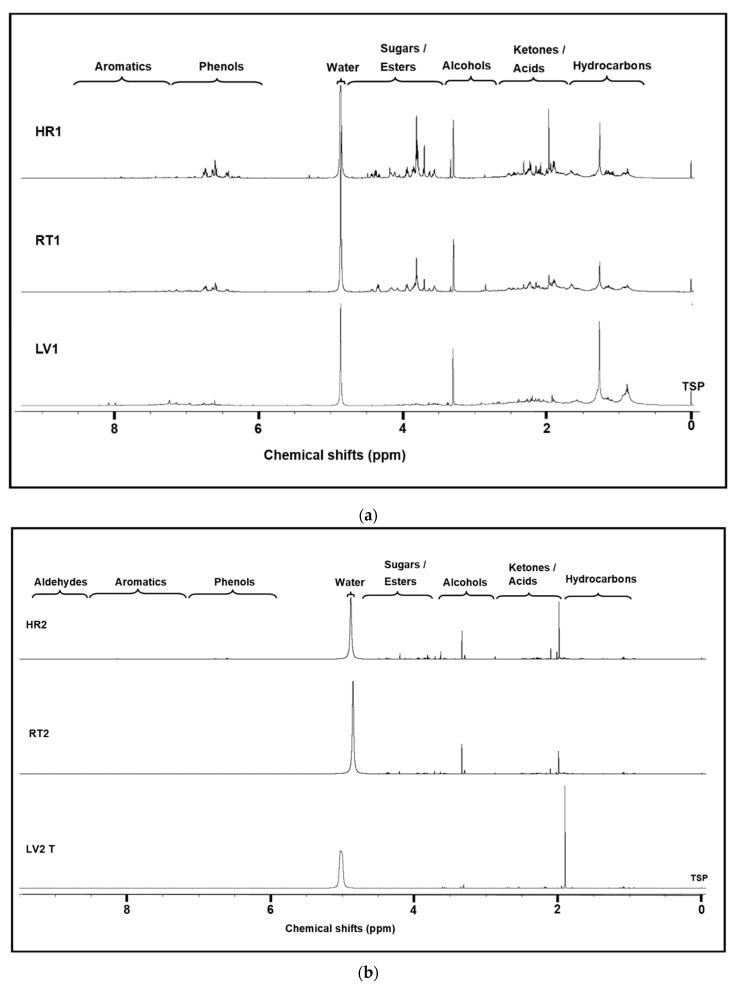
(**a**) Comparison of the proton nuclear magnetic resonance (^1^H NMR) spectra of hemp distillates pyrolyzed and condensed at temperatures 275–350 °C and 130 °C, respectively. (**b**) Comparison of the ^1^H NMR spectra of hemp distillates pyrolyzed and condensed at temperatures 275–350 °C and 70 °C, respectively.

^1^H NMR is an excellent tool to determine types of functionalities present in a sample and concentrations of assigned individual compounds. Bio-oils (LV1, HR1, and RT1) were analyzed for the concentration (mM) of the main compound groups while water-based solutions (LV2, HR2, and RT2) were analyzed for the concentration of the most abundant individual compounds. The ^1^H NMR results in six samples are shown in Figure 2a,b, LV1, HR1, and RT1 (condensed at 130 °C). The number of protons (^1^H) used to calculate the concentration depend on the number of chemically equivalent protons in each integrated signal [39]. The chemical shift regions and concentrations of different compound groups are listed in Table 2.

LV2, HR2, and RT2 (Figure 2b) condensed at 70 °C contained organic acids, alcohols, and phenols. Note that the sample LV2 consisted of two phases which were separated into two different samples, namely LV2B (bottom/oily phase) and LV2T (top/aqueous phase). The amount of LV2B was around 10% of the total LV2 composition and exhibited a similar NMR spectrum as LV1 and is not included in Figure 2b. The chemical shift regions and concentrations of the identified compounds are listed in Table 2 and Table 3.

#### 3.3.2. NMR

From the ^1^H NMR spectra and concentration values, it is evident that there are significant differences in the concentrations and chemical compositions as is highlighted in the spectra shown in Figure 2a,b. In Figure 2a (C1 category), these differences are evident especially in the chemical shift regions of ketones/acids, anhydrosugars/esters, and phenolics. In this category, as expected, LV1 had the highest concentration of hydrocarbon compounds (12.4 mM), the most upfield NMR spectrum region. HR1 and RT1 had lower and relatively similar concentration values, i.e., 6.9 mM and 8.6 mM, respectively. In general, hurd contained the highest concentration of alcohols, esters/anhydrosugars, and phenols. However, leaves had the highest concentration of ketones/acids and aromatics.

Category 2 samples, LV2T, HR2, and RT2, were analyzed so that major and minor individual compounds could be identified; as expected, these distillates mainly contained water, acetic acid, and methanol. The concentrations of the identified compounds varied as follows: acetic acid (50–241 gL^−1^), methanol (2–30 gL^−1^), propanoic acid (5–20 gL^−1^), methyl acetate (1–7 gL^−1^), and formic acid (1–2 gL^−1^). However, one ketone compound, 1-hydroxybutan-2-one (2 gL^−1^), and furans such as furfural (2 gL^−1^) and HMF (4 gL^−1^) were detected only in the HR2 distillate, revealing the major compound composition differences between the hemp leaves, hurds, and roots.

The findings of this study can be compared with the results for hurds in an earlier study [24] to give overall insight into the chemical composition of the hemp plant (leaves, hurds, and root) pyrolysis liquids. Both studies revealed that hemp leaves (aqueous phase) had the highest concentration of acetic acid 241 gL^−1^, hurd between 47–151 gL^−1^, and roots 66 gL^−1^ at operation and condensation temperatures of 275–350 °C and 70 °C, respectively. Furthermore, according to [24], pyrolysis liquids of FINOLA and FUTURA 75 (winter retted) hurd produced at operation and condensation temperatures of 135–275 °C and 70 °C had acetic acid concentrations of 259 gL^−1^ and 260 gL^−1^, respectively. The results are similar to acetic acid concentration from hemp leaves of 241 gL^−1^. The findings of this study showed that pyrolysis liquid of hemp leaves obtained at pyrolysis stage 275–350 °C and condensation temperature at 70 °C have similar chemical concentrations of acetic acid when compared to pyrolysis liquids obtained from hemp hurds at torrefaction stage 135–275 °C and the condensation temperature at 70 °C studied earlier.

#### 3.3.3. GC-MS Analysis

The results of the peak identification were accepted when a chemical match (similarity index) was equal to or greater than 90%. The GC-MS analysis was carried out with a view to compare the compounds present in both water-based solutions and bio-oil samples. Both the water-based solutions and bio-oil samples were complex mixtures of compounds such as organic acids, alcohols, ketones, phenols, sesquiterpenes, aromatic ketones, fatty acids, fatty acid methyl esters, aromatic esters, and others which are listed in Table 4, Table 5, Table 6, Table 7, Table 8, Table 9 and Table 10.

The GC-MS analysis of the bio-oil revealed different total amounts of compounds: LV1 (12 compounds), HR1 (29 compounds), and RT1 (21 compounds). The 12 compounds detected in LV1 included cannabinoids, aromatics and carbohydrates, alkenes, and alkanes, as well as some others. The most abundant compound, resorcinol, (−)-(*E*)-2-*p*-mentha-1,8-dien-3-yl-5-pentyl-, present at 6.5% is a synonym for (−)-trans-cannabidiol, a non-psychoactive compound with putative health benefits [40]. The other cannabinoids found including cannabinol CBN (mildly psychoactive compound), accounted for 2.4%, and delta-8-tetrahydrocannabinol for 0.92%. Other compounds detected at significant composition percentages were pentadecanoic acid,14-methyl-,methyl ester (4.7%), 4-ethylphenol (3.0%), and eicosane (2.16%); the remaining compounds were present at lower composition percentages.

Out of the 31 compounds identified in HR1, 13 compounds had a concentration higher than 1%. The most abundant compounds were phenols such as 2,6-dimethoxyphenol (9.7%) and 2-methoxyphenol (4.5%). Other compound groups such as sugars, furans, alcohols, cyclic ketones, and others were present at lower composition percentage values. There were 23 compounds identified in RT1; 12 of these were evident at a composition value higher than 1% and of these the vast majority (11/12) were phenols with the most abundant compounds being 2,6-dimethoxyphenol (16.6%), 2-methoxyphenol (5.1%), *p*-cresol (4.0%), 4-ethyl-2-methoxyphenol (4.0%), and cis-2-methoxy-4-propenylphenol 2.89%. HR1 and RT1 revealed similarities in their chemical compositions, i.e., a significant number of methylated or methoxylated phenolic compounds were detected including the two most abundant compounds, 2,6-dimethoxyphenol and 2-methoxyphenol. In contrast, cannabinoids were the most abundant in LV1.

According to [41], the hemicellulose content of hemp leaves was 254 mg g^−1^, cellulose 96 mg g^−1^, lignin 166 mg g^−1^, and protein 247 mg g^−1^ while fresh hemp stalk contained hemicelluloses 269 mg g^−1^, celluloses 396 mg g^−1^, lignin 218 mg g^−1^, and proteins 35 mg g^−1^. The mass concentration of lignin is known to decrease in leaves [42]. Therefore, the decrease in the amount of cellulose and lignin in LV1 is one of the major factors responsible for the low concentration of phenols in LV1. In addition, the biosynthesis of unique cannabinoid compounds takes place mainly in the glandular trichomes in the leaves of the hemp plant [43]. This accounts for the presence of cannabinoids such as THC, CBD, etc. in LV1 and explains the major chemical composition differences when LV1 is compared to HR1 and RT1.

The water-based solution fractions revealed a total of 5, 23, 26, and 28 different compounds as listed in LV2 T, LV2B, HR2, and RT2, respectively. The two most abundant compounds identified in the LV2 T were acetic acid (39.0%) and propionic acid (1.1%). LV2B had a similar chemical composition as LV1. HR2 appeared to have a phenol-predominant composition, with the two most abundant compounds being 2,6-dimethoxyphenol (7.9%) and 2-methoxyphenol (6.7%). Other phenols, ketones and furans were present at lower percentages. In RT2, 11 compounds had a composition value higher than 1% with acetic acid (11.6%), 2-methoxyphenol (9.7%), and 2,6-dimethoxyphenol (2.2%) as the three most abundant compounds. This group of phenols might be valuable for different applications and are present at high enough concentrations to allow purification.

The 2D GC-MS analysis and identification of volatile compounds in Futura 75 pyrolysis liquids (top phase and bottom phase) was performed and the results are reported in detail in [24]. The top aqueous phase, which is similar to the water-based fraction in this study, revealed the presence of monophenols such as 1,4-benzenediol, 2-methyl-1,4-benzenediol, apocynin, and 4-methylsyringol while fatty acids with their methyl esters, alkanes, alkenes, methoxyphenols and steroid compounds were detected in the bottom oily phase with 2,6-dimethoxyphenol as the most abundant compound. Compounds such as 4-methylsyringol were observed in both the top and bottom phases, e.g., in LV1 and LV2B where some compounds such as delta.8-tetrahydrocannabinol and 2,4-dimethylphenol were detected. The same situation occurs with 2,6-dimethoxyphenol and 2-methoxyphenol in HR1 and HR2. These results revealed that hemp leaves are the most suitable part of the hemp plant for producing cannabinoids and hurds and roots are more suitable parts for producing phenols. In general, all parts of hemp plants are good sources of organic acids (acetic acid and propanoic acid) especially leaves with the highest concentration.

Some investigators have reported that the pyrolytic distillate product from lignocellulosic biomass constituents such as water-based distillate (wood vinegar) is rich in phenols [44,45]; these compounds as well as acetic acid have antibacterial and antifungal activities [45,46]. According to some studies, acetic acid, 2-methoxyphenol (guaiacol), and 2,6-dimethoxyphenol (syringol) are the major compounds present in wood vinegar with pesticide, insecticide, and herbicidal activities [47,48,49]. Furthermore, several water-soluble mono- and oligophenols such as 2-methoxyphenol, 2,6-dimethoxyphenol, and 2-methylphenol (cresol) are the major phenols that are present in the bio-oil distillate and these have been reported to have significant antifungal activity as pesticides [45,50,51]. The presence of these active compounds in the industrial hemp distillate fractions, at such high concentrations, indicates that hemp distillates have similar or even more potent antibacterial, antifungal, and other pesticide properties when compared to many other lignocellulosic biomasses.

#### 3.3.4. (+) APPI FT-ICR MS

**Figure 3:**
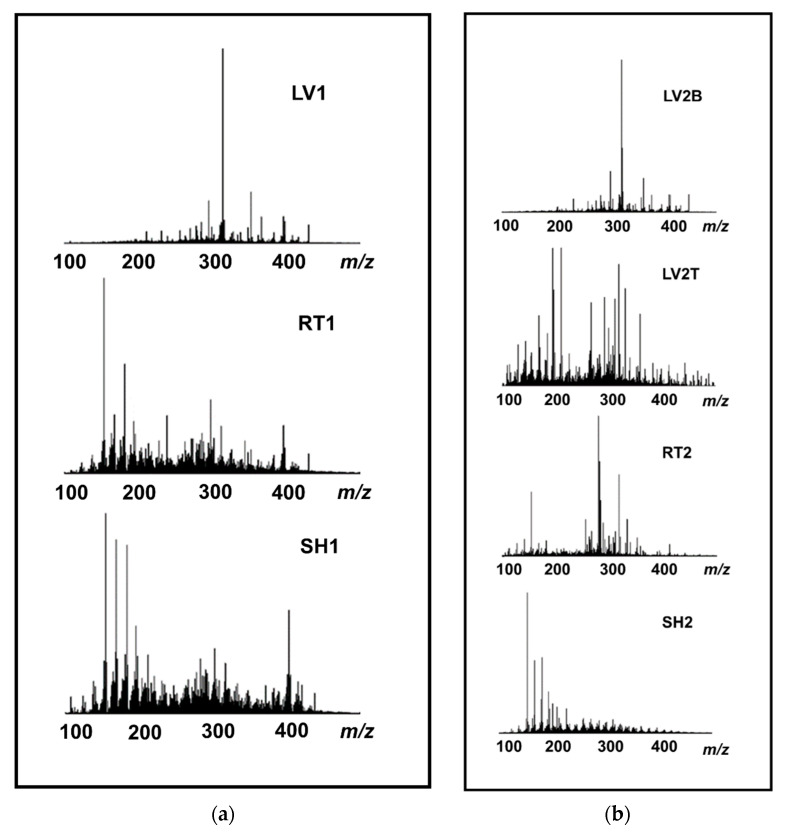
(**a**) APPI FT-ICR mass spectra of LV1, RT1, and HR1 of hemp distillates at pyrolysis stage 275–350 °C and condensed at 130 °C. (**b**) APPI FT-ICR mass spectra of LV2B, LV2T, RT2, and HR2 of hemp distillates at pyrolysis stage 275–350 °C and condensed at 70 °C.

**Figure 4:**
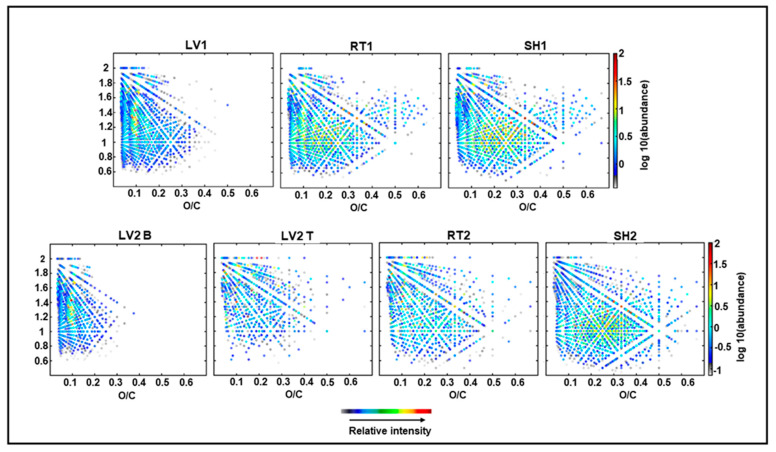
Van Krevelen diagrams (color-coded for relative intensity) for the O_x_ compounds detected in the pyrolysis oil distillates LV1, RT1, and SH1 condensed at 130 °C and pyrolysis water-based distillates LV2B, LV2T, RT2, and SH2 condensed at 70 °C by APPI FT-ICR MS.

Positive-ion APPI FT-ICR mass spectrometry was used to acquire an overall chemical fingerprinting of the distillate samples. This technique makes possible the detection of much heavier compounds as compared to GC-MS and consequently these two techniques provide a more comprehensive view of the chemical composition of the slow pyrolysis liquids. The measured (+) APPI FT-ICR mass spectra are presented in Figure 3a,b. Van Krevelen diagrams revealed the O_x_ compounds detected in the pyrolysis oil samples as shown in Figure 4. The most abundant compounds found in the distillate sample are listed in Table 11 and Table 12.

Note the mass spectra are plotted as line spectra showing all assigned peaks within *m*/*z* 100–500 at S/N ratio ≥ 6. The number of the identified CHNOS species in the samples varied from 2.400 (RT2) up to 8.300 (LV1), highlighting the very complex chemistry of the distillates.

In general, hydrocarbons (HC), oxygen-containing compounds (O_x_), nitrogen-containing (N_y_), and both oxygenated nitrogen-containing (N_y_O_x_) and sulfur-containing (O_x_S_z_) species were detected in the samples. The HC species are likely to have resulted from the extensive degradation of terpenoids. In addition, other reactions, such as dealkylation and condensation, can occur during slow pyrolysis, forming polyaromatic hydrocarbons (PAH) from lignin-derived phenols. The O_x_ class dominated in RT and HR; this class includes different types of oxygenated compounds. The hemp leaves contain more nitrogen than the other parts of the plant [41] and this can also be seen in the results; the N_y_ and N_y_O_x_ compounds were detected at a higher abundance in the leaf samples as compared to the hurds and roots.

Table 11 and Table 12 present the most abundant nonvolatile compounds found in the distillate sample. Based on the MS data, cannabidiol (C_21_H_30_O_2_) was the most abundant compound in LV1 (Table 11) and this is consistent with the GC results shown in Table 4. In addition, different terpenoids and terpenes were also found at high concentration percentages in LV1. The results of LV2B (oily phase) were very similar to LV1 (Table 12). With respect to the most abundant compounds in LV2T (aqueous phase), we detected some currently unidentified N_2_O_2_ class compounds and some O_x_ class species. In RT1, HR1, and HR2, the most abundant compound was syringol (C_8_H_10_O_3_), which results from the decomposition of lignin. Amide derivates (fatty amides) dominated in RT2 (Table 12).

Van Krevelen diagrams (i.e., atomic H/C versus O/C ratio for each compounds) for all O_x_ species are shown in Figure 4 [52]. These diagrams provide an overview of the chemical compounds present in the samples. The pyrolysis liquids produced from leaves (LV1 and LV2B) were clearly different from the root and hurd samples. LV1 and LV2B contained fewer oxygenated and more condensed species as compared to RT and HR. The most abundant compounds in LV1 and LV2B could be found in the region of H/C ≈ 1.1–1.5 and O/C ≈ 0.1, which indicates that they are likely terpenoid derivatives. The aqueous phase (LV2T) included more oxygenated and fewer aromatic species than the other LV samples, as expected.

Pyrolytic lignins, i.e., phenolic compounds (H/C ≈ 0.6–1.4 and O/C ≈ 0.2–0.5), were present at a higher abundance in RT and HR as compared to LV1 and LV2B. There were no clear differences between RT1 and HR1. In contrast, RT2 included more aliphatic and fewer lignin-derived species than HR2. No carbohydrates were detected; this is due to the fact that their efficient ionization with (+) APPI is not possible.

Despite good reputation of GC-MS and FT-ICR MS in bio-oil analysis, they have common limitations such as unavailability of mass spectra of some pyrolysis liquid compounds in MS libraries, and lack of analytical standards. These factors mean GC-MS and FT-ICR MS methods provide only partial information about the chemical composition and result in incomplete chemical composition of pyrolysis liquids. Therefore, they are not suitable for determining concentration. All these limitations are consistent with other studies [53]. On the other hand, NMR is good for determination of the concentration of compounds but its limitations such as overlapping peaks makes it difficult to determine minor compounds (compounds in low concentration) and therefore provide incomplete composition results. In general, NMR, GC-MS, and FT-ICR MS provided a complementary and comprehensive characterization of the pyrolysis liquids.

### 3.4. The Economic Assessment

The economic assessment is of great importance due to present situation in the EU and Africa concerning demand and optimal utilization of slow pyrolysis for biopesticides, biostimulants, and biofertilizers. The economic assessment of hemp pyrolysis liquids can be evaluated based on the demand for a product in society. For example, in the developing countries in Africa and Asia, the economic assessment is based on demand for cheap natural pesticides and biochar with higher heating values. This implies that the use of slow pyrolysis process would represent a promising method for the sustainable utilization of the local lignocellulose biomass which could be subjected to pyrolysis for bio-based commercial applications [18,54]. In Europe, the commercial situation is different. The economic assessment in Europe could be divided into three categories: biofuels (production of bioethanol, biodiesel, biogas) [55], biopesticides, and green chemicals [47].

If one inspects the results from a bioeconomical and financial point of view, then it is evident that the optimal utilization of water-based solutions and bio-oils from the pyrolysis process to acquire biopesticides would involve lower costs and more straightforward processes. For example, the water-based solution distillate only requires filtration and dilution and the production of a biopesticide from a bio-oil distillate simply involves dilution, centrifugation, and filtration [56,57,58]. In the future, the driving force behind the development of novel biopesticides will be the legislation on what kind of materials can be used as pesticides. There is a goal to reduce the use of synthetic pesticides in order to minimize the risks to human health and the environment (EFSA 2019). For example, the adoption of the European commission strategy in 2006 with the implementation of its directives in 2009 and most importantly the EU ban on propiconazole, the active substance in a plant protection product 2018 (EC No 396/2005, EC No 1107/2009, and commission implementing decisions (EU) 2020/27) have resulted in a high demand for natural pesticides such as those originating from liquid distillates as a replacement for synthetic chemical pesticides (EU Regulation 2016/2031).

Despite the clear need for natural, affordable pesticides and the well-known pesticide properties of plant-based liquid distillates, they have not been approved for commercial sale in Europe. This failure can be traced to the limited number of studies investigating the concentration profile of the water-based and bio-oil distillates. Further studies are needed to determine concentration profiling of lignocellulosic biomass distillates as well as testing their effects on health and the environment. Only then will it be possible to reveal the full economic potential and the possible commercialization of pyroligneous acids and bio-oils as viable biopesticide products not only in Europe but throughout the wider world.

Bio-oil is a good source of green and phytochemicals and their extraction from bio-oil is an important way to enhance the commercial value of any biochemical-related business. Related to the health and chemical industries, the separation of cannabinoids such as CBD and THC and the terpenes from the bio-oil distillate was demonstrated in this study. These compounds have potential medicinal properties, and they can be obtained by various techniques including conventional distillation. However, the separation of heat-sensitive compounds or some compounds with high boiling points requires the use of a vacuum distillation known as molecular distillation at low temperature with a short contact distillation path in an evaporator [59,60]. Phenols extracted from bio-oil distillates can be used as raw materials in the development of green chemicals. They are renewable and can be readily separated with solvents to obtain value-added products with the possibility of commercial scale production [61,62]. The extraction methods for phenols include solvent, steam, and molecular distillation. In economic terms, a solvent extraction process is a less capital-intensive process when compared to a short molecular distillation method or other types of high-tech stream extraction processes with a subsequent liquid–liquid extraction using water, ethyl acetate, pentane, or toluene.

The separation of acetic acid from pyrolysis liquids has been performed using different methods in which a typical method was liquid–liquid extraction with aliphatic tertiary amines [63]. Envisaging the economy of a separation process, distillation is the most widely used method of separating fluid mixtures and is considered to be attractive for obtaining acetic-acid-rich fractions from pyrolysis liquids. Though acetic acid was one of the main compounds from the aqueous fraction, its yield would be about 40 kgton^−1^ with a value less than EUR 100 as a bulk product with low purity. The remarkable difference between hemp leaves, hurds, and roots is the presence of 1-hydroxybutan-2-one which can only be found in the hemp hurds. It is considered as the most expensive of the compounds quantified and the total amount in the distillates is roughly 1.3 kgton^−1^ (including torrefaction and pyrolysis distillates). In general, this could be purified to higher than 95% purity and if we assume 50% efficiency, the price of product would be EUR 1300–6500 for 1 ton of raw material input with the price EUR 20–100/ g^−1^ (Molport, 2021). Based on the above rough estimates, there is clearly a potential for high value products based on hemp liquids, especially hemp hurds liquid. However, more investigations are needed to evaluate the overall potential slow pyrolysis process that can compete with other methods and be profitable commercially without incentives.

## 4. Conclusions

The detailed chemical characterization of volatile and nonvolatile compounds and assessment of the concentration of the main compound groups present in the slow pyrolysis distillates from industrial hemp leaves, hurds, and roots were successfully carried out with more than 115 compounds being identified. The stepwise slow pyrolysis with fractional condensation turned out separate aqueous and bio-oil fractions without extra steps. The process parameters such as heating rate, process, and condensing temperatures can be optimized to increase the yield of a certain compound or compound group. The aqueous fraction is mainly composed of acetic acid, methanol, propanoic acid, formic acid, furfural, and 1-hydroxybutan-2-one while the bio-oil fraction is mainly composed of fatty acids, phenols, levoglucosan, triterpenes, cannabidiol, and others.

NMR, FT-ICR MS, and GC-MS spectroscopy were successfully applied in this comparative study. The possible use of bio-oil in the chemical and agrochemical industries requires a detailed understanding and analysis of the concentrations of the individual higher molecular weight compounds present in the bio-oil. Further studies will be needed to elucidate the structures of the higher molecular compounds as well as their stability and concentration in the bio-oil. Future work should include testing of the antifungal and antibacterial efficacy of the liquid distillates and bio-oils obtained from industrial hemp leaves and roots, as well as an assessment of their commercial potential.

## Figures and Tables

**Table 1:** Slow pyrolysis process runs and mass balance of distillates.

Parameters		Fresh Hemp Sample		
		LV	HR	RT
Raw material at the beginning	(g)	12,934	5830	12,409
Liquid released in compression pressure 20 MPa	(g)	6152	1248	6125
Raw material in the process	(g)	6782	4582	6284
Dry raw material in the process Moisture content	(dry weight%)	106	135	130
Density at the beginning of process	(g/cm^3^)	1.14	0.77	1.06
Retention time	(h)			
Drying stage, DS	(h)	20	19	20
Torrefaction stage, TS	(h)	19	20	20
Pyrolysis stage, PS	(h)	4	5	6
Mass of distillates	(g)			
Drying stage, DS (25–135 °C)				
Condensation, 130 °C	(g)	13	8	57
Condensation, 70 °C	(g)	1275	893	1256
Condensation, 5 °C	(g)	501	353	474
Total mass drying stage	(g)	1789	1254	1787
Torrefaction stage, TS (135–275 °C)				
Condensation, 130 °C	(g)	24	15	43
Condensation, 70 °C	(g)	1765	913	1455
Condensation, 5 °C	(g)	441	386	531
Total mass torrefaction stage	(g)	2230	1314	2029
Pyrolysis stage, PS (275–350 °C)				
* Condensation, 130 °C	(g)	46	6	16
* Condensation, 70 °C	(g)	30	27	31
Condensation, 5 °C	(g)	44	83	81
Total mass pyrolysis stage	(g)	120	116	128
Residual char	(g)	1446	1011	1285
Mass loss	(g)	1110	887	963
Total mass yield of distillates	(%)	47	43	48
Residual char	(%)	29	30	29
Mass loss	(%)	22	27	21

* means selected samples for chemical characterization with several complementary techniques.

**Table 2:** Concentration of compound groups in hemp distillates of leaves, hurds, and roots at a condensation temperature of 130 °C by ^1^H NMR.

Chemical Shift (ppm)	Compound Group	C1 (mM)		
		LV 1	HR1	RT1
0.8–1.8	Hydrocarbons	12.4	6.9	8.6
1.8–2.9	Ketones/Acids	10.1	8.61	8.5
3.2–3.9	Alcohols **	1.5	4.7	3.9
4.2–5.0	Esters/Sugars *	0.8	8.8	5.7
6.0–7.2	Phenols	4.9	7.5	6.3
7.2–8.5	Aromatics	2.1	0.9	1.1

* signifies that a water peak was excluded, ** CD_3_OD peak excluded.

**Table 3:** Concentration of identified compounds in hemp distillates of leaves, hurds, and roots at a condensation temperature of 70 °C by ^1^H NMR.

Chemical Shift (ppm)	Compound	C2 (gL^−1^)		
		LV2T	HR2	RT2
1.99	Acetic acid	241	779	1106
3.33	Methanol	2	30	27
1.04	1-Hydroxybutan-2-one	-	2	-
1.09	Propanoic acid	20	5	11
3.64	Methyl acetate	7	1	5
8.20	Formic acid	-	0.8	0.6
9.40	HMF	-	4	-
9.56	Furfural	-	2	-

- signifies that compound was not detected.

**Table 4:** Chemical composition of distillates of hemp leaves (LV1) from pyrolysis stage (PS) condensed at 130 °C and analyzed by gas chromatography–mass spectrometry (GC-MS).

No	Compound	RT (min)	Similarity Index	Mol. Form.	Relative Abundance (%)
1	Resorcinol, (−)-(*E*)-2-*p*-mentha-1,8-dien-3-yl-5-pentyl-	44.368	98	C_21_H_30_O_2_	6.5
2	Pentadecanoic acid, 14-methyl-, methyl ester	38.847	96	C_17_H_34_O_2_	4.7
3	4-Ethylphenol	21.104	93	C_8_H_10_O	3.0
4	Cannabinol	46.549	98	C_21_H_26_O_2_	2.4
5	Eicosane	39.810	93	C_20_H_42_	2.1
6	Methyl dehydroabietate	43.533	96	C_21_H_30_O_2_	1.8
7	*p*-Cresol	18.195	95	C_7_H_8_O	1.8
8	Heptadecanoic acid, 14-methyl-, methyl ester	41.287	93	C_19_H_38_O_2_	1.1
9	Pentadecane	30.128	96	C_15_H_32_	0.9
10	DELTA.8-Tetrahydrocannabinol	45.254	92	C_21_H_30_O_2_	0.9
11	Hexadecane	32.499	93	C_16_H_34_	0.8
12	2,4-Dimethylphenol	20.502	96	C_8_H_10_O	0.3

Similarity index ≥ 90.

**Table 5:** Chemical composition of hemp hurds (HR1) distillates from pyrolysis stage (PS) condensed at 130 °C and analyzed by GC-MS.

No	Compound	RT (min)	Similarity Index	Mol. Form.	Relative Abundance (%)
1	2,6-Dimethoxyphenol	26.350	96	C_8_H_10_O_3_	9.7
2	2-Methoxyphenol	18.427	97	C_7_H_8_O_2_	4.5
3	1,4:3,6-Dianhydro-alpha-d-glucopyranose	22.270	91	C_6_H_8_O_4_	4.0
4	Creosol	21.805	97	C_8_H_10_O_2_	3.9
5	3-Methoxy-1,2-benzenediol	23.832	96	C_7_H_8_O_3_	3.8
6	4-Ethyl-2-methoxyphenol	24.349	91	C_9_H_12_O_2_	3.7
7	2-Methoxy-4-(1-propenyl)phenol	28.897	95	C_10_H_12_O_2_	2.2
8	Hexadecanoic acid, methyl ester	38.842	98	C_17_H_34_O_2_	2.1
9	*p*-Cresol	18.100	95	C_7_H_8_O	2.1
10	3-Ethyl-2-hydroxy-2-cyclopenten-1-one	19.464	96	C_7_H_10_O_2_	1.5
11	Maltol	19.223	90	C_6_H_6_O_3_	1.4
12	3-Methyl-1,2-cyclopentanedione	16.164	95	C_6_H_8_O_2_	1.4
13	Catechol	22.171	91	C_6_H_6_O_2_	1.2
14	2,6-Dimethoxy-4-(2-propenyl)phenol	34.810	95	C_11_H_14_O_3_	0.9
15	4-Ethylphenol	21.087	91	C_8_H_10_O	0.8
16	1-Heptacosanol	41.966	94	C_27_H_56_O	0.5
17	Docosanoic acid, methyl ester	45.525	99	C_23_H_46_O_2_	0.5
18	9-Octadecenoic acid, methyl ester, (*E*)-	41.071	96	C_19_H_36_O_2_	0.4
19	Heptadecane	42.027	95	C_17_H_36_	0.3
20	Methyl stearate	41.282	99	C_19_H_38_O_2_	0.3
21	2,3-Dimethoxytoluene	23.225	96	C_9_H_12_O_2_	0.3
22	4-Methyl-1,2-benzenediol	24.912	92	C_7_H_8_O_2_	0.2
23	1,2,3-Trimethoxy-5-methylbenzene	27.765	96	C_10_H_14_O_3_	0.2
24	Methyl 18-methylnonadecanoate	43.257	98	C_21_H_42_O_2_	0.2
25	2,3-Dimethyl-2-cyclopenten-1-one	16.581	90	C_7_H_10_O	0.2
26	Tricosanoic acid, methyl ester	47.040	93	C_24_H_48_O_2_	0.2
27	Heneicosane	40.977	98	C_21_H_44_	0.1
28	Eicosane	39.810	95	C_20_H_42_	0.1
29	Methyl 13-methyltetradecanoate	37.284	90	C_16_H_32_O_2_	0.1

Similarity index ≥ 90.

**Table 6:** Chemical composition of hemp roots (RT1) distillates from pyrolysis stage (PS) condensed at 130 °C and analyzed by GC-MS.

No	Compound	RT (min)	Similarity Index	Mol. Form.	Relative Abundance (%)
1	2,6-Dimethoxyphenol	26.350	97	C_8_H_10_O_3_	16.6
2	2-Methoxyphenol	18.432	94	C_7_H_8_O_2_	5.1
3	*p*-Cresol	8.130	95	C_7_H_8_O	4.0
4	4-Ethyl-2-methoxyphenol	24.353	90	C_9_H_12_O_2_	4.0
5	cis-2-Methoxy-4-propenylphenol	28.910	96	C_10_H_12_O_2_	2.8
6	Creosol	21.818	96	C_8_H_10_O_2_	1.8
7	Phenol	14.688	95	C_6_H_6_O	1.7
8	4-Ethylphenol	21.095	91	C_8_H_10_O	1.6
9	Homovanillyl alcohol	30.954	81	C_9_H_12_O_3_	1.4
10	Hexadecanoic acid, methyl ester	38.847	99	C_17_H_34_O_2_	1.2
11	3-Ethylphenol	21.160	90	C_8_H_10_O	1.2
12	Phenol, 4-(2-propenyl)-2,6-dimethoxy	34.819	94	C_11_H_14_O_3_	1.1
13	2,3-Dimethylphenol	20.532	92	C_8_H_10_O	0.9
14	2,3-Dimethylcyclopent-2-en-1-one	16.590	90	C_7_H_10_O	0.8
15	3-Ethyl-2-hydroxy-2-cyclopenten-1-one	19.477	96	C_7_H_10_O_2_	0.8
16	2-Methylphenol	17.382	95	C_7_H_8_O	0.7
17	4-Ethyl-3-methylphenol	23.221	90	C_9_H_12_O	0.6
18	Methyl dehydroabietate	43.533	96	C_21_H_30_O_2_	0.5
19	2-Methoxy-4-(1-propenyl)phenol	27.869	91	C_10_H_12_O_2_	0.4
20	2,4-Dimethylphenol	20.493	93	C_8_H_10_O	0.3
21	2-Methoxybenzenethiol	24.004	92	C_7_H_8_OS	0.3

Similarity index ≥ 90.

**Table 7:** Chemical composition of distillates of hemp leaves (LV2T) from pyrolysis stage (PS) condensed at 70 °C and analyzed by GC-MS.

No	Compound	RT (min)	Similarity Index	Mol. Form.	Relative Abundance (%)
1	Acetic acid	2.707	91	C_2_H_4_O_2_	39.0
2	Propanoic acid	10.242	90	C_3_H_6_O_2_	1.1
3	1,4:3,6-Dianhydro-alpha-d-glucopyranose	4.364	90	C_6_H_8_O_4_	0.5
4	2-Furanmethanol	7.178	97	C_5_H_6_O_2_	0.3
5	3-Methyl-2-cyclopenten-1-one	16.568	93	C_6_H_8_O	0.1

Similarity index ≥ 90.

**Table 8:** Chemical composition of distillates of hemp leaves (LV2B) from pyrolysis stages (PS) condensed at 70 °C analyzed by GC-MS.

No	Compound	RT (min)	Similarity Index	Mol. Form.	Relative Abundance (%)
1	Cannabidiol	44.368	99	C_21_H_30_O_2_	8.9
2	Hexadecanoic acid, methyl ester	38.842	99	C_17_H_34_O_2_	4.5
3	Eicosane	39.810	98	C_20_H_42_	3.2
4	Phenol	14.645	94	C_6_H_6_O	2.8
5	Phytol	41.145	91	C_20_H_40_O	2.4
6	Cannabinol	46.541	99	C_21_H_26_O_2_	2.3
7	Caryophyllene	28.179	99	C_15_H_24_	2.2
8	Octadecane	48.275	95	C_18_H_38_	1.8
9	DELTA.8-Tetrahydrocannabinol	44.424	93	C_21_H_30_O_2_	1.6
10	4-Ethylphenol	21.095	93	C_8_H_10_O	1.5
11	4-Methylphenol	18.113	96	C_7_H_8_O	1.2
12	Humulene	29.044	97	C_15_H_24_	1.1
13	2,4-Dimethylphenol	20.514	91	C_8_H_10_O	0.9
14	Pentadecane	30.128	97	C_15_H_32_	0.8
15	Methyl stearate	41.282	99	C_19_H_38_O_2_	0.8
16	*p*-Cymene	16.030	97	C_10_H_14_	0.8
17	Methyl dehydroabietate	43.529	99	C_21_H_30_O_2_	0.7
18	Toluene	4.351	94	C_7_H_8_	0.7
19	Undecane	18.862	96	C_11_H_24_	0.7
20	Methyl 10-trans,12-cis-octadecadienoate	40.942	99	C_19_H_34_O_2_	0.7
21	Dronabinol	45.573	99	C_21_H_30_O_2_	0.7
22	1-Pentadecene	29.939	97	C_15_H_30_	0.6
23	Tetradecane	27.628	97	C_14_H_30_	0.5

Similarity index ≥ 90.

**Table 9:** Chemical composition of hemp hurds (HR2) distillates from pyrolysis stage (PS) condensed at 70 °C and analyzed by GC-MS.

No	Name	RT (min)	Similarity Index	Mol. Form	Relative Abundance (%)
1	2,6-Dimethoxyphenol	26.359	96	C_8_H_10_O_3_	7.9
2	2-Methoxyphenol	18.432	97	C_7_H_8_O_2_	6.7
3	3-Methylcyclopentane-1,2-dione	16.194	95	C_6_H_8_O_2_	3.1
4	Creosol	21.806	97	C_8_H_10_O_2_	2.7
5	4-Ethyl-2-methoxyphenol	24.353	91	C_9_H_12_O_2_	2.2
6	Maltol	19.318	94	C_6_H_6_O_3_	2.1
7	2-Cyclopenten-1-one	6.873	91	C_5_H_6_O	1.9
8	3-Ethyl-2-hydroxy-2-cyclopenten-1-one	19.521	96	C_7_H_10_O_2_	1.6
9	3-Methoxy-1,2-benzenediol	23.858	97	C_6_H_8_O_2_	1.5
10	2-Furanmethanol	8.341	97	C_5_H_6_O_2_	1.0
11	3-Methyl-2-cyclopenten-1-one	13.543	94	C_6_H_8_O	1.0
12	Phenol	14.645	94	C_6_H_5_OH	0.9
13	1-(2-Furanyl)ethanone	11.134	91	C_6_H_6_O_2_	0.8
14	2,3-Dimethyl-2-cyclopentenone	16.564	90	C_7_H_10_O	0.6
15	2-Methyl-2-cyclopenten-1-one	10.837	91	C_6_H_8_O	0.5
16	4-Ethylphenol	21.083	94	C_8_H_10_O	0.5
17	Eugenol	26.522	94	C_10_H_12_O_2_	0.3
18	Phenol, 2-methyl-	17.369	96	C_7_H_8_O	0.2
19	2-Methylphenol	15.097	90	C_7_H_8_O	0.2
20	2,6-Dimethoxy-4-(2-propenyl)phenol	32.624	97	C_11_H_14_O_3_	0.1
21	2-Methoxy-4-(1-propenyl)phenol	27.860	90	C_10_H_12_O_2_	0.1
22	3,4-Dimethoxytoluene	23.230	96	C_9_H_12_O_2_	0.1
23	2-Furanmethanol, acetate	15.050	90	C_7_H_8_O_3_	0.1
24	1,2,3-Trimethoxy-5-methylbenzene	27.770	96	C_10_H_14_O_3_	0.1
25	1,3-Dimethyl-1-cyclohexene	15.880	87	C_8_H_14_	0.1

Similarity index ≥ 90.

**Table 10:** Chemical composition of hemp root (RT2) distillates from pyrolysis stage (PS) condensed at 70 °C and analyzed by GC-MS.

No	Compound	RT (min)	Similarity Index	Mol. Form.	Relative Abundance (%)
1	Acetic acid	2.724	91	C_2_H_4_O_2_	11.6
2	2-Methoxyphenol	18.462	94	C_7_H_8_O_2_	9.7
3	2,6-Dimethoxyphenol	26.384	96	C_8_H_10_O_3_	2.2
4	3-Methylcyclopentane-1,2-dione	16.306	95	C_6_H_8_O_2_	2.1
5	1-(2-Furyl)ethanone	11.099	91	C_6_H_6_O_2_	1.9
6	4-Hydroxybutanoic acid	11.508	90	C_4_H_8_O_3_	1.8
7	Butyrolactone	11.434	90	C_4_H_6_O_2_	1.6
8	2-Furanmethanol	8.543	97	C_5_H_6_O_2_	1.4
9	2-Cyclopenten-1-one	6.881	91	C_5_H_6_O	1.4
10	3-Methyl-2-cyclopenten-1-one	13.582	95	C_6_H_8_O	1.4
11	Propanoic acid	3.972	90	C_3_H_6_O_2_	1.3
12	Phenol	14.739	93	C_6_H_6_O	1.2
13	2,3-Dimethyl-2-cyclopenten-1-one	16.581	93	C_7_H_10_O	1.0
14	Creosol	21.818	97	C_8_H_10_O_2_	0.9
15	2-Methyl-2-cyclopenten-1-one	10.797	94	C_6_H_8_O	0.9
16	4-Ethyl-2-methoxyphenol	24.357	90	C_9_H_12_O_2_	0.5
17	5-Methyldihydro-2(3H)-furanone	13.181	91	C_5_H_8_O_2_	0.5
18	Methyl 4-hydroxybutanoate	14.593	90	C_5_H_10_O_3_	0.4
19	2-Methoxy-5-methylphenol	21.603	93	C_8_H_10_O_2_	0.4
20	2-Methylphenol	17.399	97	C_7_H_8_O	0.3
21	4-Ethylphenol	21.117	94	C_8_H_10_O	0.3
22	Cyclopentanone	5.121	90	C_5_H_8_O	0.3
23	Tetrahydro-2H-pyran-2-one	17.115	90	C_5_H_8_O_2_	0.2
24	3-Methoxy-1,2-benzenediol	23.948	97	C_7_H_8_O_3_	0.2
25	2-Methoxytetrahydrofuran	4.058	93	C_5_H_10_O_2_	0.1
26	2,4-Dimethylphenol	20.519	96	C_8_H_10_O	0.1
27	3-Methyl-2(5H)-furanone	14.279	90	C_5_H_6_O_2_	0.1
28	3-Butyl-2-hydroxy-2-cyclopenten-1-one	25.239	95	C_9_H_14_O_2_	0.1

Similarity index ≥ 90.

**Table 11:** Most abundant compounds detected in LV1, RT1, and HR1 with APPI FT-ICR MS.

*m*/*z*	Molecular Formula	Compound *	Relative Abundance (%)
**LV1**			
314	C_21_H_30_O_2_	Cannabidiol	100
352	C_24_H_32_O_2_	Unknown	25
295	C_20_H_22_O_2_	Diterpenoid	21
366	C_25_H_34_O_2_	Sesterterpene	13
396	C_29_H_48_	Triterpene hydrocarbon	13
398	C_29_H_50_	Triterpene hydrocarbon	11
285	C_19_H_24_O_2_	Diterpenoid	11
430	C_29_H_50_O_2_	Triterpenoid	9
**RT1**			
154	C_8_H_10_O_3_	2,6-Dimethoxyphenol	100
182	C_10_H_14_O_3_	Dihydroconiferyl alcohol	56
298	C_18_H_18_O_4_	Unknown	31
168	C_9_H_12_O_3_	Homovanillyl alcohol	30
239	C_18_H_22_	Triterpene hydrocarbon	30
396	C_29_H_48_	Triterpene hydrocarbon	25
312	C_19_H_20_O_4_	Unknown	24
**HR1**			
154	C_8_H_10_O_3_	2,6-Dimethoxyphenol	100
168	C_9_H_12_O_3_	Homovanillyl alcohol	87
182	C_10_H_14_O_3_	Dihydroconiferyl alcohol	85
396	C_29_H_48_	Triterpene hydrocarbon	52
194	C_11_H_14_O_3_	Vanillyl acetone	44
298	C_18_H_18_O_4_	Unknown	32

* Tentative peak assignments: Pubchem, Phenol Explorer, Lipid MAPS.

**Table 12:** Most abundant compounds detected in LV2B, LV2T, RT2, and HR2 with APPI FT-ICR MS.

*m*/*z*	Molecular Formula	Compound *	Relative Abundance (%)
**LV2B**			
314	C_21_H_30_O_2_	Cannabidiol	100
295	C_20_H_22_O_2_	Diterpenoid	27
352	C_24_H_32_O_2_	Unknown	22
430	C_29_H_50_O_2_	Triterpenoid	12
396	C_29_H_48_	Triterpene hydrocarbon	11
278	C_18_H_30_O_2_	Pinolenic acid	11
366	C_25_H_34_O_2_	Sesterterpene	11
231	C_15_H_18_O_2_	Cyclohexylcinnamate	9
**LV2T**			
211	C_11_H_18_N_2_O_2_	Unknown	100
195	C_10_H_14_N_2_O_2_	Unknown	99
319	C_22_H_38_O_1_	Unknown	88
331	C_19_H_38_O_4_	Glyceryl palmitate	70
292	C_18_H_28_O_3_	Unknown	64
312	C_20_H_24_O_3_	Diterpenoid	63
267	C_18_H_34_O_1_	Octadecanal	60
359	C_21_H_42_O_4_	Glyseryl stearate	52
169	C_8_H_12_N_2_O_2_	Unknown	51
**RT2**			
280	C_18_H_33_N_1_O_1_	Hexadecanamide	100
282	C_18_H_35_N_1_O_1_	Octadecenamide	68
319	C_22_H_38_O_1_	Unknown	58
154	C_8_H_10_O_3_	2,6-Dimethoxyphenol	46
284	C_18_H_37_N_1_O_1_	Octadecanamide	40
334	C_22_H_39_N_1_O_1_	Unknown	26
256	C_16_H_33_N_1_O_1_	Hexadecenamide	26
**HR2**			
154	C_8_H_10_O_3_	2,6-Dimethoxyphenol	100
182	C_10_H_14_O_3_	Dihydroconiferyl alcohol	54
168	C_9_H_12_O_3_	Homovanillyl alcohol	52
194	C_11_H_14_O_3_	Vanillyl acetone	29
202	C_13_H_14_O_2_	Unknown (phenolic)	21
210	C_11_H_14_O_4_	Sinapinyl alcohol	19
228	C_15_H_26_O_2_	Bisphenol A (impurity?)	17

* Tentative peak assignments: Pubchem, Phenol Explorer, Lipid MAPS.

## Sample Availability

Samples of the compounds are not available.

## Abbreviations

EIHA	European Industrial Hemp Association
t·ha^−1^	Tonne per hectare
HR	Hemp hurds
LV	Hemp leaves
RT	Hemp hurds, and Hemp roots
(LV1, HR1, RT1)	#1 refers to bio-oil (oily) pyrolysis liquid
(LV2, HR2, RT2)	#2 refers to aqueous (water-based) pyrolysis liquid
NMR	nuclear magnetic resonance spectroscopy
GC-MS	gas chromatography-mass spectrometry
FT-ICR MS	Fourier transform ion cyclotron resonance mass spectrometry
h	hours
